# Perspective Mapping: Tutorial for Collecting Quantifiable Qualitative Interview Data

**DOI:** 10.2196/72622

**Published:** 2025-08-14

**Authors:** Jennifer Mammen, Mirinda Tyo, Sandhya Seshadri

**Affiliations:** 1 College of Nursing and Health Sciences, University of Massachusetts Dartmouth, 285 Old Westport Rd, North Dartmouth, MA, 02747, United States, 1 5089998586; 2 Department of Neurology, University of Rochester, Rochester, NY, United States

**Keywords:** online interviewing, mind mapping, mixed methods, patient experience, digital data, qualitative, digital technology, research methods

## Abstract

Mixed methods research is essential to development of patient-reported outcome measures, digital technology, and endpoint selection for clinical drug trials and to advance clinical care when complex health-related experiences cannot be fully understood by quantitative or qualitative approaches alone. New technology and opportunities for remote data collection have changed the ways in which qualitative and quantitative data can be collected, enabling researchers to capture human experiences in ways not previously possible. This paper describes Perspective Mapping, a new online interviewing technique that uses mind mapping software to capture in-depth qualitative data inside a quantitative measurement framework to understand and measure individual experiences. The objective of this tutorial is to review the theoretical underpinnings, present instructions for study design and implementation, and address strengths, limitations, and potential applications of this technique in health and behavioral sciences. During videoconferencing interviews, mind-mapping software is used to visually depict experiences. Structured concept maps are cocreated in real time with participants, focusing on building detailed narrative descriptions about experiences and categorizing these within a predefined quantitative framework, such as the relative importance of different experiences relevant to a phenomenon. The approach combines semistructured interviewing with technology-enhanced card-sorting techniques, allowing participants to define and prioritize what matters most. This method ensures narrative richness alongside structured data collection, facilitating deeper understanding of phenomena. Perspective Mapping emphasizes participant engagement in data generation and analysis and enables the simultaneous collection of qualitative narratives and quantitative assessment of key concepts. The variations of the technique have been successfully applied in research on chronic illness, symptom burden, and digital health technology. Advantages of the approach include systematic collection of qualitative data, transparent and structured data outputs, real-time data validation, and the ability to return maps to participants as a form of reciprocity. Feasibility factors, such as interviewer capabilities, participant literacy, interview duration, and technology resources must be considered. Perspective Mapping offers an innovative and engaging way to gather complementary qualitative and quantitative data remotely. By blending qualitative depth with quantitative structure, the technique supports richer, more actionable insights for health research, policy, and beyond. This technique holds promise for applications in health, psychology, education, and other social sciences where comprehensive understanding of experiences is essential.

## Introduction

### Background

Mixed methods research can be used to answer questions about complex health phenomena insufficiently understood by quantitative or qualitative approaches alone [1,2]. While randomized controlled trials have historically been viewed as the gold standard for health research, mixed methods are increasingly being recognized as equally necessary and mutually beneficial [3]. This paradigm shift has been accelerated by recent Federal Food and Drug Administration guidance indicating that patient perspectives and transparent demonstration of “meaningfulness” are needed for regulatory approval of new drugs, devices, and clinical outcomes assessments [4-7].

Qualitative research methods help to uncover the what, why, and how of health-related phenomena. This involves “asking the expert” in order to understand the individual’s experiences, perceptions, and behaviors within real-life settings [8,9]. Guba [10] referred to this as “naturalistic inquiry,” with the assumption that there may be multiple realities in real-world settings. Thus, data collection is “discovery oriented” and prioritizes interviews, focus groups, or open-response surveys. By contrast, quantitative research methods are based on a rationalistic paradigm and assumptions of a single reality that can be objectively studied. Therefore, it is more oriented toward answering questions about quantities (eg, prevalence, frequency, severity, and duration) and changes in quantities due to other factors, such as time, treatments, or behavioral interventions. For this reason, quantitative research generally focuses on evaluation of phenomena under controlled or manipulated conditions, with measurement of predefined concepts of interest (eg, opinions, experiences, and biomarkers) via standardized instruments and other outcome measures [11].

Quantitative and qualitative approaches each have strengths and weaknesses that have been reviewed and debated extensively [12-14]. Common concerns with quantitative methods include the underlying assumptions that knowledge of the phenomena is adequate to determine valid constructs for measurement, over-reliance on large sample sizes to ensure validity, decreased awareness of important interpersonal variations, and depersonalization of findings [15]. Qualitative research, by contrast, has been criticized as not broadly generalizable due to smaller sample size, lack of probability sampling, fluid or personalized data collections approaches (unstandardized), and data outputs that are not easily amenable to numeric summations (eg, transcripts and quotes) [11,16-20].

For all of these reasons, there is a growing trend toward using mixed methods or blended approaches to answer complex, multifaceted research questions, particularly with regard to interventions, treatments, and health policies [21]. However, gaps between current methods and desired objectives remain, particularly with regards to measuring personal experiences in ways that are feasible and support broader generalizability of findings. Internet-based research offers a unique opportunity to address this need. Emerging capabilities of digital technology and opportunities for remote data collection have changed the ways in which qualitative and quantitative data can be collected. Specifically, new software and technology can enable researchers to capture human experiences in ways not previously possible. In this paper, we describe a new mixed methods technique for videoconferencing interviewing called “Perspective mapping” that uses mind mapping software to support in-depth, qualitative exploration of personal experiences in ways that are transparently quantifiable. We first discuss the theoretical underpinnings, then present step-by-step instructions for design and implementation, and conclude by discussing strengths, limitations, and possible applications of perspective mapping for health and behavioral sciences.

### Theoretical Underpinnings

Perspective mapping is a computer-based technique in which an interviewer and a participant jointly create a visual representation of the participant’s experiences using mind mapping software via videoconferencing during an online interview [22]. During a mapping interview, a single phenomenon is explored in depth, using a mind map as a visual tool to identify key concepts and the experiences that relate to these. Experiences and perceptions are then reorganized inside a quantitative framework that measures a particular attribute, such as the relative importance to the person. Any type of perspective or experience can be explored, for example, bothersome symptoms, important factors contributing to quality of life, the impact of chronic illness on patients, caregiver experiences, student educational experiences, or the usability and feasibility of new digital technologies and workflows [22,23]. The underlying commonality, irrespective of focus, is that perspective maps are extensive, ordered, diagrammatic representations of the experiences, views, beliefs, or values of an individual about something that they have personally experienced.

This application of mind mapping is a digital extension of prior paper-based card sorting techniques, which have been used for years in qualitative research to understand how participants categorize or prioritize experiences [24]. The advantage of using software via videoconferencing interview is the ability to build extensively detailed maps of experiences in real-time, remotely [25,26]. Theoretically, mapping aligns with Symbolic Interactionism and Constructivist theory [27], which hold that reality, including meaning, understanding, and knowledge, is the product of interactions between individuals and is thus “co-constructed” [28,29]. The orientation toward co-created meaning is at the heart of the mapping process, and the goal is to achieve shared understanding by representing experiences in terms that are understandable to the interviewer, participant, and others beyond the interview.

Methodologically, this would be considered a convergent mixed methods approach, as quantitative+qualitative data are collected side-by-side to explore a single phenomenon [21]. An interviewer conducts an in-depth interview with a single participant. Experiences described by the participant are concisely transcribed by the interviewer into the map in the form of “nodes.” Multiple nodes are (1) strung together to build a descriptive summary of a single concept relevant to the phenomenon and (2) multiple concepts are organized within a categorical quantitative framework (eg, nominal or ordinal) to create ordered groupings. This process generates a branching word diagram about a phenomenon of interest that contains in-depth qualitative experiences alongside quantifiable data about concept frequency and relative priority. The concurrent qualitative exploration plus quantitative sorting and ranking enables the interviewer to discover what each person prioritizes and the ways they perceive or are affected by their experiences, as shown in Textbox 1.

Textbox 1. **Perspective Mapping Highlights.**

Perspective mapping is a hybrid qualitative+quantitative technique. It involves conducting an in-depth qualitative interview about a phenomenon of interest, identifying key concepts, creating concise visual summaries of the experience as reported by the participant, and organizing this inside of a quantitative framework for measurement. Throughout the mapping process, the interviewer and participant engage in shared data generation and analysis with iterative member checking, which contributes to validity.

## Methods

### Overview

**Figure 1.**
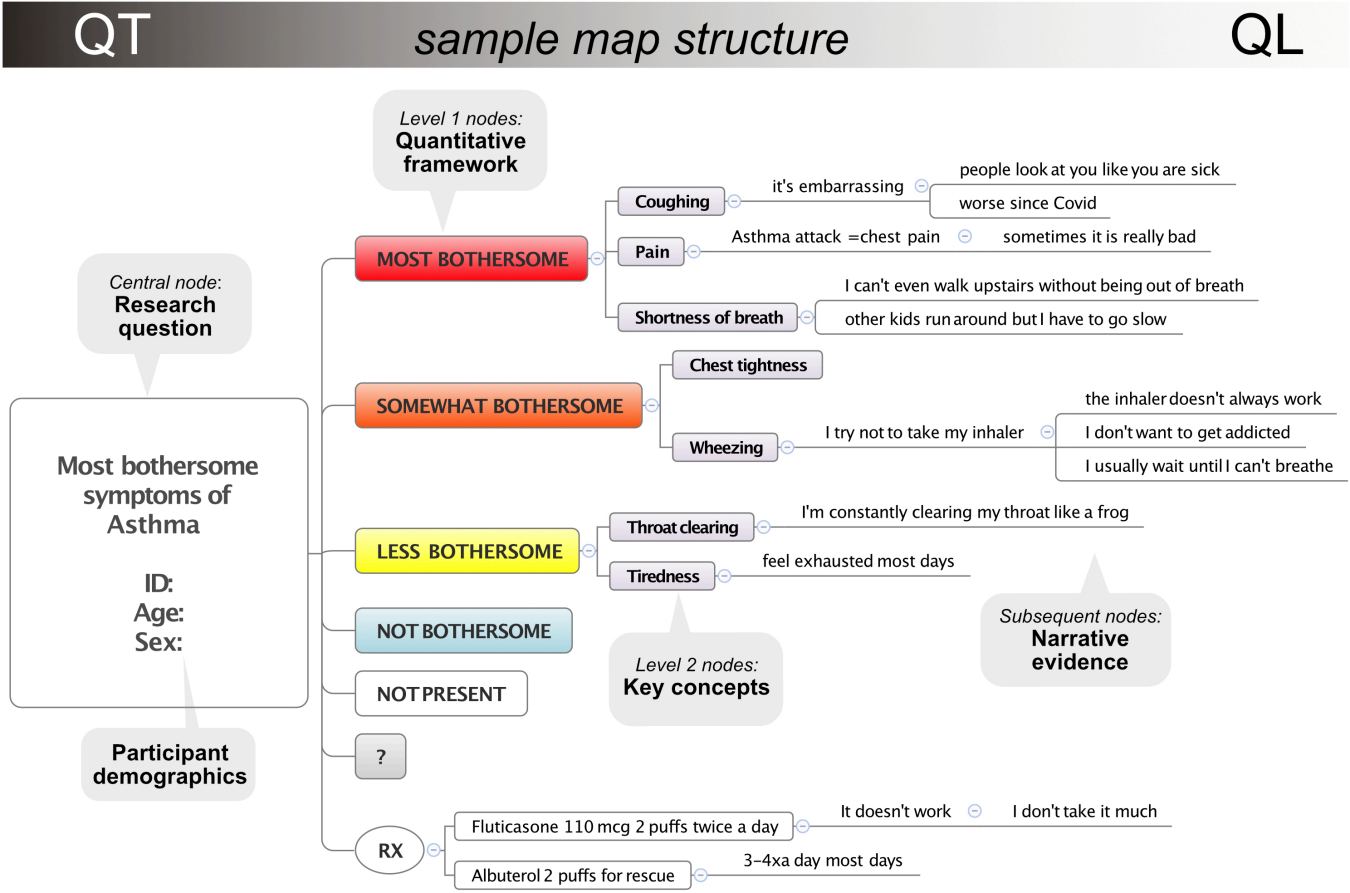
Example of map structure to explore bothersome symptoms of asthma.

Figure 1 provides an illustration of the components of a perspective map, and video demonstrations can be viewed online [30]. Perspective maps comprise a collection of concisely worded key concepts directly related to a single main topic (eg, bothersome symptoms of asthma). The concepts are nested inside a quantitative framework that supports grouping and ranking by priority. A branching network of qualitative descriptions (narrative evidence) is attached to each concept and depicts experiences with that concept. Maps are created jointly by the interviewer and participant during a videoconferencing interview using screen sharing, so that the participant is continuously directing, observing, and validating any data entered in the map.

### Planning and Preparation

**Figure 2.**
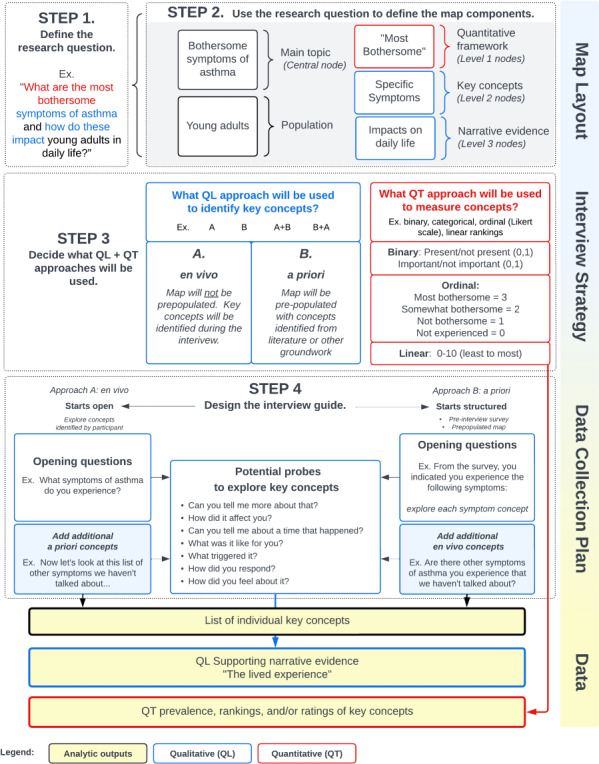
Process for designing a perspective mapping interview.

Conducting a perspective mapping interview requires substantial planning and preparation. Planning should include defining the research question and map structure, including qualitative and quantitative components, obtaining feedback from participant advisors, and training to achieve technical proficiency. This stage can take 1‐3 months to complete and is dependent on multiple factors, such as the experience and expertise of the research team, the complexity of the research question, the phenomenon of interest, and the depth of information being sought. Figure 2 delineates the process for designing a perspective mapping protocol. Step-by-step descriptions and practical considerations are discussed below.

#### Step 1. Define the Research Question

A perspective mapping research question should identify (1) the population of interest, (2) the main topic to be explored, (3) key concepts the researcher wants to know more about, and (4) the attribute to be measured (quantitative framework). In general, it is advisable to choose a single main topic, which will be explored in depth. A research question for a perspective mapping study might look like this:

**RQ**: *What are the most bothersome symptoms of asthma and how do these impact young adults in their daily life?*

The population is who will be recruited to participate (young adults with asthma). The main topic is what the interviewer and participant will talk about (symptoms and impacts of asthma). Key concepts are the specific experiences related to the main topic the researcher wants to explore and measure, which in this situation would be individual symptoms [22]. By contrast, if the main topic was “things that trigger my asthma,” key concepts would be individual triggers (eg, “catching a cold,” and “stressful situations”). “Most bothersome” implies that the researcher will quantitatively assess how much each key concept matters to the participant along a scale of relative bothersomeness. The narrative evidence is the qualitative story of how, when, where, and why the key concepts are experienced by the participant.

#### Step 2: Design the Map Components

The mind map is a participant-facing representation of the research question. It should mirror the research question components, reframed in layperson terms in a logical and aesthetically pleasing manner. The main topic (phenomenon of interest) will be the central node (Figure 1). The quantitative measurement categories for grouping information will be first-level nodes and second-level nodes will usually be key concepts. There can be variation to this schema based on study objectives. For consistency, a map template should be developed prior to data collection so that all participants are viewing and using the same basic structure. This is an essential part of the standardized approach that enables quantification. Standardization includes using consistent layout and formatting, in which quantitative categories and key concepts have a designated appearance and structure. Map templates can be created at the start of the study and duplicated for use with each participant. We use Xmind8 mapping software, which has a free version that is sufficient for most studies. The “Pro” version is useful for designing permanent templates with custom features [31]. Tutorials for creating maps are available on the developer website, including how to edit the map and make it visually appealing, which is an important aspect of the participant experience [32].

#### Step 3: Decide the Qualitative and Quantitative Approaches

##### Qualitative Approach to Concept Elicitation

The heart of mapping is the semistructured interview, which is used to identify and explore key concepts relevant to the phenomenon of interest. Key concepts should be short, succinct labels [32]. For example, “wheezing” might be a key concept for bothersome symptoms of asthma. Key concepts are typically given their own unique shape and color (eg, yellow with a thick border) to make them stand out visually, which is helpful to the participant and simplifies data analysis for the researcher. These are most commonly located so that when reading down a map vertically, the concepts will appear immediately adjacent to the quantitative framework and read like an ordered list (Figure 1).

Mapping interviews are semistructured, with three basic approaches to exploring key concepts: (1) en vivo, (2) a priori, and (3) a combination of both (Figure 2). With an en vivo approach, the interviewer starts without any predetermined concepts and asks the participant to identify concepts that are personally important. The benefit of this approach is that the interview is entirely participant-led and prioritizes what matters to the person at that time. However, this approach has disadvantages. Not all participants will experience all concepts, and not all people experiencing a concept will find it important enough to recall or mention. Therefore, greater variation will occur in concept coverage during interviews. With an exclusively en vivo approach, it is only possible to measure what people spontaneously reported experiencing. It is not possible to say whether they do or do not have certain experiences, as can be done with an a priori approach. An example of an en vivo approach would be starting an interview with a blank map containing only the quantitative framework (eg, most to least important) and asking the participant to identify what matters most to their personal quality of life related to a specific disease.

With an a priori approach, the interviewer starts out with a predetermined and often extensive list of concepts they want to know about. These concepts can be derived from literature, pre-interview surveys (eg, checklists), expert knowledge, or other relevant groundwork. For example, the participant might be asked to identify concepts that are relevant to them personally from a checklist. During the interview, the interviewer would then systematically explore each concept identified. Participants often find the a priori approach easier because they do not have to come up with the concepts on the spot and can use the checklist as a starting point. While this is more comprehensive, the a priori approach has the potential to introduce bias by being overly leading and can risk prioritizing normative or mainstream views. An a priori approach is generally most useful when there are specific concepts that must be explored and evaluated or when a comprehensive assessment is needed. An example of an a priori approach would be administering a preinterview survey to gather initial information about the phenomenon of interest (eg, a literature-based checklist or validated instrument) and using the information derived from the survey to identify key concepts for exploration during the interview.

A third option is to use a combination of a priori and en vivo approaches—either starting open-ended and then adding any concepts not spontaneously volunteered (A+B) or starting with pre-existing concepts and asking participants to identify any that are missing (B+A). For example, an interviewer could begin by allowing a participant to freely identify what is most important to them and then conclude by asking them to consider a remaining list of concepts not already covered. Alternately, the interviewer could begin by using a concept checklist and then asking the participant to identify if any concepts are missing that they believe are also important. This dual approach enables the interview to be maximally participant-driven, while still supporting a comprehensive assessment. We have used the spontaneous+structured approach (A+B) in much of our work [22,23,33].

##### Quantitative Framework

The quantitative framework comprises the predetermined groupings that will be used for ranking and sorting key concepts. These can be any nominal or ordinal categories that align with the research question. Examples of nominal categories would be “barriers” and “facilitators,” which, translated to lay terms, might be represented as “things that help” and “things that hinder.” Examples of ordinal categories include time frames “early disease,” “mid-stage disease,” “late disease,” etc., or Likert scales, such as “most” to “least” important. The quantitative approach must be decided before conducting interviews, as the same framework should be used for all interviews to support valid comparisons. While there may be situations where a quantitative framework is not desired, it is important to note that without a preexisting measurement framework, the ability to quantify the data will be limited.

Ranking and sorting are done during the interview, with the participant directing the process. This can occur while exploring experiences or after all experiences have been identified and discussed. Key concepts are sorted into the measurement framework by the interviewer as directed by the participant. For example, if the participant indicates that the key concept of coughing is what bothers them the most, that node would be moved to the top of the “most bothersome” category in the map. Further hierarchical sorting may also be useful to rank and prioritize concepts within individual quantitative categories. Quantification is an iterative process, with the option to reorganize the structure multiple times as the interview progresses. As a note, when sorting and ranking, all narrative evidence should be collapsed (hidden from view) to allow the participant to focus only on the ranking of key concepts.

### Narrative Evidence

A perspective map should tell a story that is understandable by someone who was not present during the original interview. It should read like an abbreviated story, which is done by adding narrative evidence. Narrative evidence consists of detailed qualitative descriptions of the individual’s personal experiences and perceptions about each key concept. For a key concept of “coughing,” supporting narrative evidence might be direct quotes from the participant, such as “it’s embarrassing” followed by “people look at you like there is something wrong with you when you start coughing,” the latter of which explains why the participant experiences embarrassment as a result of coughing. In the map, narrative evidence is added adjacent to the concept and can be expanded or hidden from view as needed to facilitate discussion and control the amount of information on the screen. This evidence is represented on screen in a visually less dominant way—such as smaller text or as a simple line. Narrative evidence follows a branching logic structure, with clusters of free text nodes grouped in a way that tells a coherent story when reading across the map. This might require iterative reorganization during the interview, as participants often do not tell their stories in a linear fashion. Thus, it is advisable to build narrative evidence as one idea per node to maximize the ability to quickly reorganize. This means the interviewer will be distilling information, creating summaries of experiences, and analyzing data on the spot, and organizing the data to tell a coherent story. It is important to emphasize it will not be feasible to capture every word. However, the interviewer should try to concisely and accurately represent as much as possible, retaining the participant’s voice. This means maintaining first-person tense and using the participants' words verbatim while eliminating nonsubstantive words and redundancy. This can be challenging, especially for clinicians who have been trained to medicalize stories for clinical documentation. A good approach is to avoid third-person statements (“he said”) and profession-specific language, unless the participant used those terms. For example, instead of stating, “this makes him feel stigmatized,” put what the participant said—“people look at me like I’m drunk.”

It is important to note that there will be instances in which adding narrative evidence to create a comprehensive perspective map is neither desirable nor feasible. For example, people with lower literacy or cognitive impairments might find it overwhelming and distracting to have data entered in real-time on screen. Similarly, not all interviewers will be comfortable simultaneously talking, typing, and reorganizing. In both situations, real-time data entry has the potential to diminish rather than enhance the interview. Pretesting to ensure that it is important to pretest to ensure that the approach is suitable for the context. The research team should be prepared to adjust approaches as needed to create an interview that is comfortable for both the participant and interviewer. In such situations, a simplified mapping approach may be preferred, in which only the key concepts are entered on screen, without further narrative evidence attached. Once the discussion is completed for a concept, it can be sorted into the quantitative category and hidden away. The simplified approach is faster, easier, and minimizes on-screen activity. Qualitative details that explain the quantitative sorting and ranking are later extracted from either the transcript or video after the interview has concluded. Other adaptations might include using more than one interviewer, allowing one to focus on interview questions and the other to type responses.

### Step 4. Design the Interview Guide

The interview guide is designed to elicit core concepts and experiences about concepts. Developing a detailed interview protocol is important to promote consistency among interviews, reduce errors in data collection, and minimize missing data about key concepts. However, to capture the individual participant’s insights, it is important that there is also room to explore. A semistructured interview guide provides a minimum set of questions that should be asked during the interview along with the approximate order of questions, while retaining flexibility to expand and adapt as needed. Having a few main questions will allow for a more in-depth and focused exploration and will yield better data. Partnering with people from the community of interest or advisory boards is useful for obtaining feedback about the interview questions, approach, and map design. Pilot testing with experts, peers, or laypersons can also help to identify items that are unclear or ambiguous, as well as provide an estimate of how long it will take to complete interviews. Multimedia Appendix 1 provides an example of a mapping interview guide to explore bothersome symptoms of rheumatoid arthritis.

### Step 5. Develop a Training Plan

A systematic training plan for interviewers will be needed to ensure technical proficiency and protocol fidelity. Depending on the complexity of the data collection plan, interviewer training will likely need to include (1) orientation to software and mapping techniques; (2) “Map along” or simulation sessions; (3) volunteer participant training interviews, and (4) debriefing after each training stage, including a comprehensive review of the work product. Map-along sessions involve watching training videos of prior mapping interviews and “mapping along” to mimic the process as conducted by an experienced interviewer. This allows the trainee to focus on the mechanical skill of data capture using the mapping software. Simulation sessions involve having one trainee act as the participant and the other as the interviewer, with emphasis on speed and accuracy of data entry. Once the initial stages have been navigated successfully, trainees should conduct several full-length interviews with volunteers who have the condition of interest until proficiency is attained. For the first “real person” volunteer participant interview, the trainer should be present for support and technical optimization. If successful, the second volunteer participant interview can be conducted independently with retrospective review of the video to assess technical skill and fidelity to the protocol. The total number of sessions will depend on the trainee’s ability to master each stage. We find that most new interviewers need 2‐3 sessions at each stage before they are ready to collect real data. Training will help determine whether 1 or 2 interviewers are needed and whether full or simplified mapping approaches are most suitable.

### Interviewing

**Figure 3.**
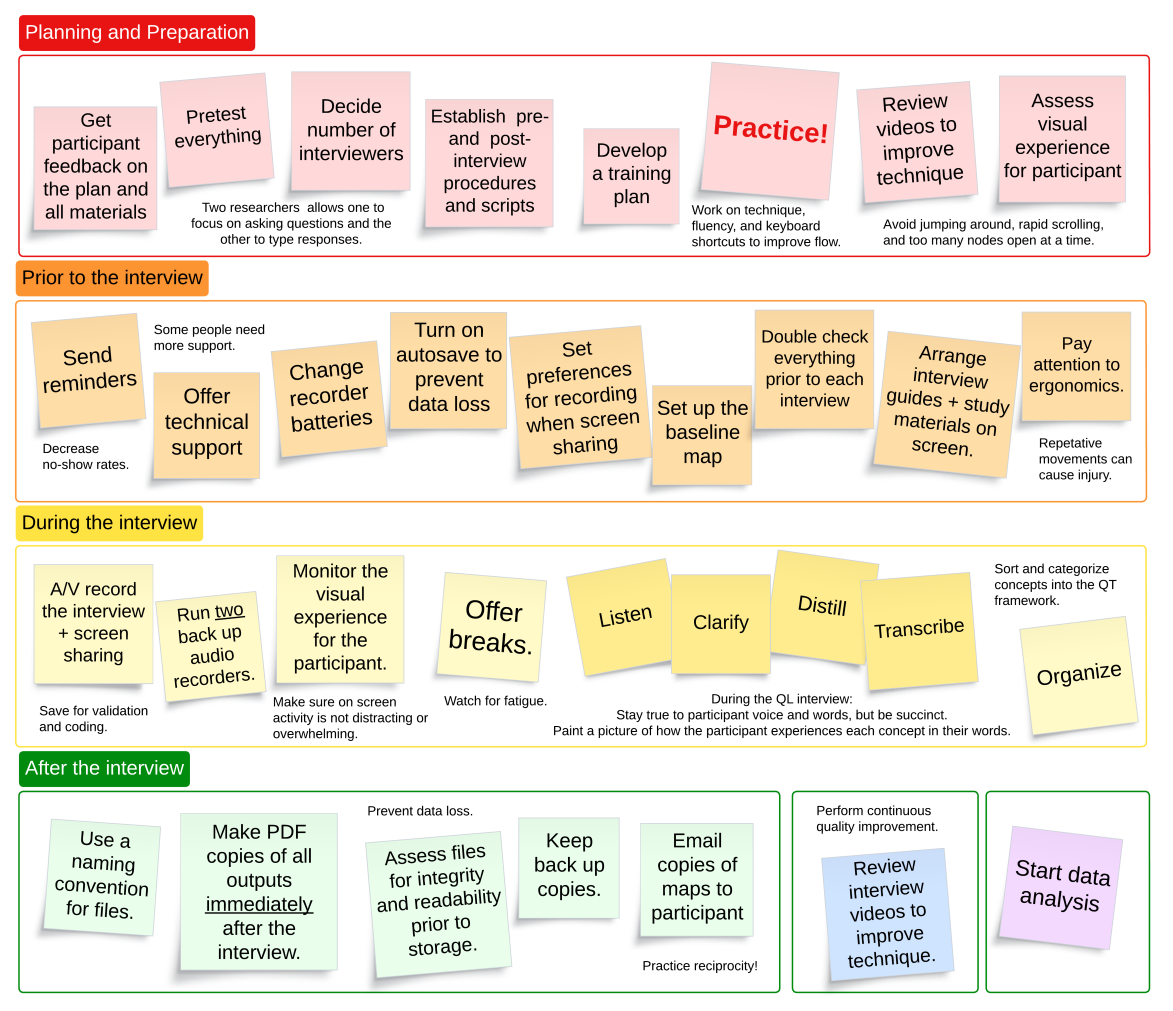
Things to consider before, during, and after a perspective mapping interview.

Figure 3 presents considerations for before, during, and after the interview. One or two weeks prior to the interview, the research team should send a reminder to the participant and include an alternate contact for technical assistance. If using an a priori approach, the researcher should create a baseline map prepopulated with the participant’s answers to the preinterview survey questions prior to the interview. It is also advisable to pretest all recording equipment, check the battery on electronic devices, turn on autosave, and edit the settings on the video-recording platform to ensure participant privacy. We generally factor half an hour on either side of an interview to accommodate setup and postinterview data management procedures.

During the interview, the goal is to create a visual representation of how the participant experiences each concept. Efforts should be focused on preserving the participant’s voice and words while ensuring brevity to minimize on-screen content. The researcher must also be mindful of the visual experience of mapping for the participant. Care is needed to ensure movements on the screen are slow, fluid, and not distracting, and that there is not too much information on the screen. This can be one of the biggest challenges for novices, who are often overly focused on entering and organizing data and less aware of the unpleasant sense of “jumping around” for the participant—akin to watching someone else scrolling for information on a computer. This is best managed by minimizing up-down and side-to-side screen movement and collapsing inactive areas of the map as soon as is reasonable. After exploring experiences in depth, the key concept should be sorted into the quantitative framework, with nodes fully collapsed before sorting.

During the interview, it is also important to monitor for signs of participant fatigue and distress and offer comfort breaks. We traditionally offer a 5‐10 minute break midway through each interview and additional breaks as needed. Some participants might struggle to verbalize experiences due to cognitive impairments or the emotional nature of the experience (eg, becoming teary over illness experiences). Strategies to manage distress include empathetic waiting, pausing or halting the interview, offering the option to skip or move on to other items, and offering the option to resume later, if desired. While some participants may elect to stop, our experience has been that most wish to share their experiences with a compassionate interviewer and value being heard even when discussing challenging topics.

After the interview has concluded, all data files should be labeled according to a clearly defined naming convention identified in the research protocol. Mapping studies generate multiple data files for each participant, often including audio, video, Xmind, PDF, and documents. A good naming convention helps to keep files organized and might include the participant ID, site, sequential order of interview, interviewer’s initials, and file type (eg, P2104_UMassD_ Int1_JM_condensedMap). Transcripts are stored as documents for qualitative analysis. Retaining audiovisual recordings may also be desirable, as this allows the research team to review for missing data or clarify wording or meaning. Reviewing videos is also a good way for newer researchers to improve interviewing techniques. Finally, maps should be converted to PDF files to preserve images for later data analysis. We converted both the collapsed (key concepts only) and fully expanded versions (key concepts+narrative evidence) to PDF . These can be emailed to participants to review for accuracy and reciprocity. We have found that being offered a PDF copy of their personal perspective map is a strong attraction for many participants [22]. When converting to PDF in Xmind, we use the “Print” function and save as a PDF, which has generated the best output. Xmind also offers several other export options.

### Analysis

Shared data analysis starts during the interview as the interviewer and participant distill information and quantitatively sort concepts together. After the interview, a second level of analysis is performed by the research team. This includes content coding of maps and identification of themes from transcripts. In this section, we discuss approaches to content coding perspective maps based on methods described by Hsieh and Shannon [34].

#### Coding Maps

**Figure 4.**
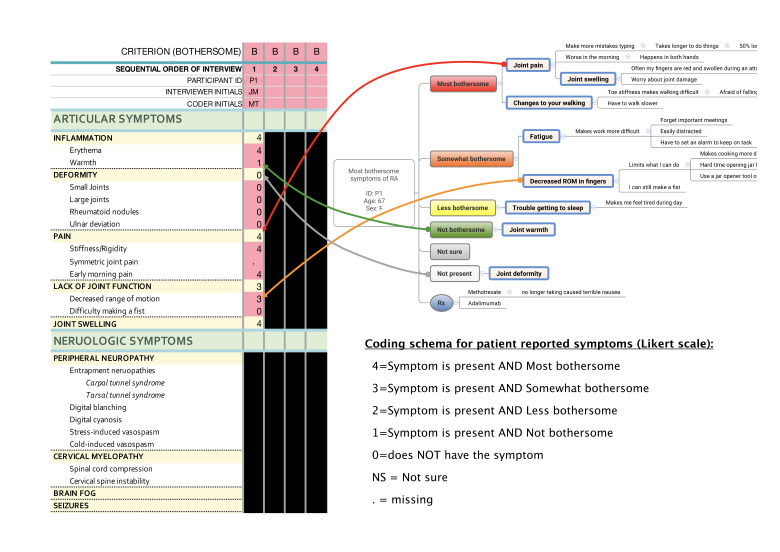
Example of a spreadsheet for content coding bothersome symptom of rheumatoid arthritis.

When coding maps, we use spreadsheets (eg, Microsoft Excel) rather than traditional qualitative data analysis (QDA) software. QDA software is our go-to resource for coding the transcripts but can be cumbersome and technically difficult to use when coding map images. Using a spreadsheet enables systematic coding of concepts and reduces the risk of coding errors, as alerts can be assigned to cells with missing values. For ease of use, our spreadsheets are organized with the participant identifiers along the top and key concepts in rows down the side, as there are typically more concepts than participants. Figure 4 presents an example of a coding spreadsheet for bothersome symptoms of rheumatoid arthritis, which is based on a Likert scale anchored to bothersomeness (0=not present, 1=present/not bothersome, 2=present/less bothersome, 3=present/somewhat bothersome, 4=present/most bothersome).

A general coding approach includes: (1) open coding of perspective maps using a spreadsheet to qualitatively identify key concepts; (2) repeat coding of maps using the spreadsheet to quantitatively determine either presence or absence (binary), categorical groupings (nominal), or hierarchical rankings (ordinal) of each key concept by participant; and (3) evaluation of transcripts to identify themes and supporting verbatim quotes. Examples of this approach are presented in our prior work [22,23,33]. The time required to conduct a data analysis of this sort depends on coder skill and depth of the analysis. In our work, we budget for 1 hour per map to code key concepts [22]. Coding narrative evidence is more variable and requires a higher level of interpretation to synthesize for commonalities across participant experiences. For this, we plan for 1‐2 hours per map for an experienced researcher and double that for a novice researcher. Thematic analysis of the transcripts can require 6‐10 hours of data analysis time for every 1‐2 hours of interview time. We often use a first coder followed by a second coder for validation; however, this is not necessary, and others have debated the merits of intercoder reliability as a measure of quality [4,35]. Use of a second coder will depend on study needs and resources. These pragmatic considerations are important to keep in mind when planning a perspective mapping study.

### Statistical Analysis

Once content coding of maps has been completed, data can be prepared for statistical analysis. In general, only simple descriptive statistics (eg, frequencies and means) will be required for cross-sectional studies with a single, group design. Studies planning for between-group comparisons or evaluating change over time in key concept priority will most likely need to use nonparametric statistics, such as McNemar or Wilcoxon signed-rank test, which can accommodate data without a normal distribution, as is often the case with Likert scale data [36].

### Transcripts

Thematic analysis and other traditional approaches to coding are suitable in this context as well. Transcripts can be derived from Zoom (Zoom Video Communications) transcription or professional services and uploaded into traditional QDA software. As with all QDA, the choice of coding approaches will depend on the underlying research question and paradigm [8,21,37]. Coding for themes and categories can be conducted in tandem with content coding of maps or independently. The researcher might elect to tell the qualitative story first and use quantitative outcomes to enhance the narrative or present quantitative data first with graphs and charts and use qualitative findings to illustrate the summaries of experiences. Examples of these types of analyses are presented in our previously published work [22,23,26,33].

## Discussion

### Principal Findings and Comparison With Previous Works

Mixed methods are important in health research due to the ability to generate deep understanding and support the robust, multifaceted assessments necessary for clinical trials and practice. While quantitative research is needed to inform policies, protocols, and treatment guidelines, qualitative research is essential to understanding psychosocial aspects of health [4,5], including “what matters” to individuals, families, and communities, and is a prerequisite to creating a culture of kind, compassionate, effective, and inclusive health care [38]. Qualitative studies illuminate phenomena and can help researchers to logically and emotionally understand the depth and scope of human experiences while highlighting non-mainstream variations. However, the inherently individualistic nature of such work makes generalization challenging, leading to questions of how best to apply findings [12]. Methodology that can cross this divide will be essential to maximizing the utility of qualitative research for health care and policy.

Perspective mapping is a technique that can help to address this gap by enabling researchers to collect detailed qualitative data in a systematic manner that is fully quantifiable. While quantification has had a disputed history in qualitative research, most experts agree that qualitative researchers can and do count, and that when used thoughtfully, inclusion of qualitative+quantitative findings offers rich insights about experiences [39,40]. Mixed methods approaches, such as described here, enable researchers to quantify without compromising the richness and dimensionality of the participant’s lived experience [38]. Thus, we believe that the approaches described here represent the start of an important next-generation “hybrid” methodology that can harness the capabilities of multi-modal online technologies to increase the power of human-centric research for mainstream application. Furthermore, due to the highly structured nature of image and textual data outputs, it is likely that mapping will integrate well with advanced analytic techniques, such as natural language processing, sentiment analysis, or other artificial intelligence–assisted data analysis (eg, extracting themes and generating interview summaries).

To date, various forms of perspective mapping have been successfully used across a range of studies exploring experiences of patients living with chronic illnesses, including asthma, dementia, Parkinson, and Huntington disease [22,24,41-45]. Most of these have focused on identifying meaningful symptoms and how individuals were impacted physically and psychosocially by symptoms. However, others have used it to explore quality of life [43] and caregivers' perceptions of important aspects of caregiving [41]. Perspective mapping has also been used to evaluate participant perceptions of new digital health technologies, which is critical to regulatory approvals [22]. Broader possibilities exist, with the potential to capture experiences across a wide range of contexts and conditions. While most examples presented in this paper have focused on chronic illness and symptom science, researchers in education, behavior, psychology, psychiatry, and other social sciences can also benefit from the use of this technique. Dyadic evaluation may represent another unique opportunity for comparison and may be particularly useful for understanding the impact of chronic conditions on patients and families.

While having many potential applications, it is important to note that this method has limitations and may be less suitable in certain contexts and with certain populations. The ability to successfully design and implement a mapping study will depend on whether the approach is truly feasible for the interviewer, participant, environment, and available resources. Conducting a mapping interview requires multitasking and computer and software proficiency on the part of the interviewer, who must be able to talk, listen, evaluate, type, and organize information in rapid succession. The participant needs access to a computer or tablet with internet access and some level of computer literacy and must be able to read information entered on the screen. People who cannot read or have visual, hearing, or cognitive impairments could find certain aspects of mapping difficult. Interviews are long and intensive and may take upwards of 2 hours, which may not be feasible for all individuals. Participants living with chronic illnesses might find it difficult to attend a 2-hour interview and may require more frequent breaks or shorter interviews. Environmental factors, such as being in a quiet place, free of distractions and noises, should also be considered, as well as access to necessary resources (eg, a computer and stable Wi-Fi). Individuals without a computer or with limited internet access could find participation difficult. People who are older, rural, or of lower socioeconomic status have historically had reduced access to computers [46], which could introduce selection bias into the sampling approaches by excluding those with limited resources. Extending the technique to smartphone use could increase demographic and geographic reach, as most of the US and global populations now have smartphones and internet access [47,48]; however, this application remains to be systematically explored. Simplified approaches (ie, sorting concepts only without narrative evidence) could be useful in these situations where the screen size is smaller, as well as for those with lower reading literacy or cognitive impairments [24].

Nonetheless, we believe that perspective mapping can offer an engaging way to conveniently and systematically collect in-depth qualitative data that reflects individual experiences in a way that is amenable to validation and summation. The emphasis on participant engagement throughout data generation, sorting, analysis, and interpretation, and the use of constant verification ensure that participants’ experiences are accurately represented [49,50]. Furthermore, iterative discussion and revision promote transparency and enhance validity [38,50]. The use of complementary data collection approaches (eg, surveys, interviews, and mapping) offers the opportunity to explore experiences from different angles (eg, triangulated data collection), with the ability to measure diverse attributes and qualitatively illustrate experiences with verbatim quotes [21,51]. Finished maps act as an audit trail and can be used to support discussion with the research team about coding decisions and thematic findings. Other strengths of the approach include co-development of interview protocols with patient panels, rigorous training procedures to ensure fidelity of data collection, ability to conduct intercoder reliability assessments, and assessment of data saturation [51]. Finally, returning PDF copies of maps to participants after the interview offers a unique opportunity for reciprocity [8,51]. Participants can share maps with health care providers and family members to track illness and communicate personal experiences. Thus, perspective mapping not only collects rich data but also offers the rare opportunity to give something meaningful back.

### Conclusions

Rigorous QL/QT data are needed to support the development of patient-reported outcome measures, digital technology, and endpoint selection for clinical drug trials and to advance clinical care. Perspective mapping is an engaging and innovative online approach to collecting complementary qualitative and quantitative data, with meaningful outputs that can be shared with participants. This technique will be relevant to diverse fields of study and a wide range of contexts.

## Supplementary material

10.2196/72622 Multimedia Appendix 1 Sample preinterview survey, interview guide, and preinterview checklist.

## Abbreviations

QDA: qualitative data analysis
