# Supplementary material for: Perspective Mapping: Tutorial for Collecting Quantifiable Qualitative Interview Data
Source: J Med Internet Res. 2025 Aug 14;27:e72622. doi: 10.2196/72622 (PMC12352805; doi:10.2196/72622)
Supplement: Multimedia Appendix 1 [file jmir-v27-e72622-s001.docx]

Multimedia Appendix 1

Section A.

Sample: Online Interview guide + survey questions

***Note:*** *The following survey questions will be administered to all participants in the Rheumatoid Arthritis (RA) qualitative study. This is a computer-based survey, which will be administered using [______] prior to the online interview.*

Introduction to Survey

The purpose of this survey is to learn about **your symptoms of Rheumatoid Arthritis**, and to what extent these changes are bothersome or important to you. We will use the list you provide as a starting point for the online interview, so greater detail is appreciated.

(Practical tip: consider writing your responses to the survey questions in another application like Microsoft word and simply cutting and pasting your answer into each box.)

Thank you for taking time to complete this survey!

Section 1. Background

[Demographic data here – link to baseline data via RA Study ID or add baseline demographics here]

1. What health conditions do you currently have?

- (Open response)

1. What medications do you currently take to manage your Rheumatoid Arthritis?

- (Open response)

Section 2. Symptoms of Rheumatoid Arthritis

1. Please tell us about the symptoms of Rheumatoid Arthritis that you experience. List each symptom one at a time and briefly describe the ways it affects you.

- (Open response)

1. Of the symptoms you described, **which** ones **are most bothersome to you** and why?

- (Open response)

Section 3. Rheumatoid Arthritis Assessment

1. Do you experience any of the following issues: (YES/NO/unsure - matrix)

| **MOVEMENT CHANGES** | **SENSORY CHANGES** | **MOOD CHANGES** |
| --- | --- | --- |
| Restlessness or hard time sitting still | Numbness or tingling in joints | Changes in mood |
| Slower movements | Joint tenderness | Feeling more anxious |
| Muscle stiffness | Pain in one joint | Worry about the future |
| Muscle cramping or spasming | Pain in 3 or more joints | Worry about being a burden on others |
| Loss of general coordination | Changes in your skin sensation | Sadness |
| Changes to your walking | Carpal tunnel (neuropathy in wrists) | Feeling depressed |
| Changes in your balance | Tarsal tunnel (neuropathy in feet) | Feelings of grief or loss |
| Decreased range of motion | Intolerance to cold temperatures | Suicidal thoughts |
| Difficulty with daily activities | **SLEEP CHANGES** | More negative feelings/emotions |
| Difficulty with cleaning or housework | Trouble getting to sleep | Obsessive-compulsive behaviors or thoughts |
| Dropping objects | Trouble staying asleep | Irritability |
| Difficulty feeding self | Poor sleep quality | Frustration |
| Difficulty with personal hygiene | **SOCIAL CHANGES** | Emotional outburst |
| Difficulty writing | Difficulty with relationships | Decreased motivation |
| Difficulty using phone or computer | Avoiding social interaction | Less interested in things |
| Difficulty making a fist | Avoidance of relationships | Changes in your personality |
| Difficulty exercising | **OTHER CHANGES** | Indifference |
| **JOINT SYMPTOMS** | Symptoms come and go (remission/exacerbation) | Decreased confidence |
| Warmth | Symptoms always present |  |
| Redness | Fever |  |
| Joint effusion (fluid in joint) | Fatigue or lack of energy |  |
| Small (finger, toes) joint changes | Rashes |  |
| Medium (neck, wrist) joint changes | Weight loss |  |
| Large (wrist, knee, hip) joint changes | Loss of appetite |  |
| Rheumatoid nodules | Dry mouth |  |
| Swelling in 3 or more joints | Itchy or dry eyes |  |
| Joint stiffness for at least one hour in the morning | Seizures |  |
| Joint swelling on one side of the body |  |  |
| Joint swelling that affects the same joints on both sides of the body |  |  |

Please note this is not a comprehensive list of symptoms.

Conclusion

This concludes the survey. Thank you for your time and for all the insightful information and experiences you have shared!

RA Interview Guide – Table of Contents

Table of Contents

[Interview procedures 5](#_Toc190343628)

[Interview scheduling 5](#_Toc190343629)

[Pre-interview Checklist 5](#_Toc190343630)

[Post interview Checklist 5](#_Toc190343631)

[RA interview filenames 6](#_Toc190343632)

[Introduction to Interview – Not recorded 6](#_Toc190343633)

[Background 6](#_Toc190343634)

[Interviewer role 7](#_Toc190343635)

[Recording begins 8](#_Toc190343636)

[Section 1. Personally Important Symptoms 8](#_Toc190343637)

[In-depth review of RA symptoms 8](#_Toc190343638)

[Ranking of symptoms by bothersomeness 9](#_Toc190343639)

[Section 2. Patient Perspectives on DHT – Debriefing on sensors 9](#_Toc190343640)

[Standardized questions for digital measures. 9](#_Toc190343641)

[Section 3: Mapping DHT to Personal RA Symptoms 10](#_Toc190343642)

[Integrate PRO Pictographs into the symptom map 10](#_Toc190343643)

[Closing questions 10](#_Toc190343644)

[Rheumatoid Arthritis Symptoms for Pre-interview Survey 12](#_Toc190343645)

RA Interview Guide – Patient

***Note:*** *Below is a semi-structured interview guide. It is to be used as a guide only. The actual areas of conversation are fluid and may be discussed at moments different from the order appearing below. The interview will be approximately 90 minutes. The interviewer may adapt the guide in order to cover the topics in the amount of time allotted for the session or in order to best elicit concepts from the participants.*

**Notes to Interviewer:**

- This interview guide is meant to help guide the discussion, but not to be used as a verbatim script; probes and questions may change slightly depending on individual feedback.
- Additional unscripted probes to be used to gain further information or clarification may include:
  - **Clarification:** I don’t quite understand that.
  - **Expressing understanding:** How did you cope with that?
  - **Justification:** Can you tell me a little bit more about why you chose that for your answer?
  - **Importance:** How important is this for you?
  - **Relationship:** I’m not sure how these 2 things are linked.
  - **Extending narrative:** Tell me a bit more about that.
  - **Accuracy:** Let’s see if I’ve got that right.

**Key for Interviewer:**

- Questions/text to be asked of the participant
- *Notes to the interviewer (Do not read to participant)*

# Interview procedures

## Interview scheduling

Schedule via HIPAA secure Zoom using the template email.

*Prior to the start of the discussion, please confirm:*

*Participant has been deemed eligible*  Yes  No

*Participant has consented prior to the discussion*  Yes  No

## Pre-interview Checklist

This will typically take ½ hour prior to the interview to complete

Confirm that the participant completed informed consent and the symptom survey in advance

Label the map and all files with the naming convention established on next page.

**Prepare your starting map** using the map template, symptom survey, screen shots of app images

Turn on autosave on Xmind.

Tech check – set up back up recorders (keep an eye on batteries as they can die mid-way

## Post interview Checklist

Add Demographics added to each map should include:

- Age
- Gender
- Years since diagnosis of RA

Print map to PDF x 4 – condensed, expanded, DHT map, CG map

Save all files to Participant folder - Use naming convention for file labels on following page

Upload complete file packet to Box. Should include the following:

one Xmind file – combines both Pt and CG

PDF of the maps (3 if no CG; 4 if CG)

Zoom audio/video recordings – both audio only and video

Copy of interview guide checklist used

Copy of completed symptom survey from Redcap

**Confirm that file transfer of all files if of good quality** **online**

**Copy** the audio only portion of Zoom to the transcription folder.

Email map to participant if requested. Send follow up thank you template email with maps attached.

## RA interview filenames

1. Source data

***Video recording – Patient (P)***

- P<subID-num>_interview<interview number (2 digits)>_<interviewer initials>_Video.<ext>
- e.g. *P001_PT_interview01_EN_Video.mp4*

***Audio recording – Patient (P)***

- P<subID-num>_interview<interview number (2 digits)>_<interviewer initials>_Audio.<ext>
- e.g. *P001_interview01_EN_Audio.mp4*

***Mindmap Xmind file – Patient +/- CG additions***

- P<subID-num>_interview<interview number (2 digits)>_<interviewer initials>_mindmap.<ext>
- e.g. *P001_interview01_EN_mindmap.xmind*
- ***Xmind file should have the following tabs****: (1) condensed symptom map, (2) expanded symptom map, (3) Full symptoms to DHT map*

1. Derived data

**#1. Condensed Symptom map (PDF)**

- P<subID-num>_interview<interview number (2 digits)>_<interviewer initials>_CondMap.<ext>
- *e.g. P001_interview01_EN_CondSymtMap.pdf*

**Expanded Symptom map (PDF)**

- P<subID-num>_interview<interview number (2 digits)>_<interviewer initials>_ExpandMap.<ext>
- e.g. *P001_interview01_EN_ExpandSymtMap.pdf*

**Full Symptom to DHT map (PDF) – Patient only**

- P<subID-num>_interview<interview number (2 digits)>_<interviewer initials>_DHTMap.<ext>
- e.g. *P001_interview01_EN_DHTMap.pdf*

**Transcript (Word doc)** **if a separate CG file, label with CG instead of P*

P<subID-num>_interview<interview number (2 digits)>_<interviewer initials>_transcript.<ext>

e.g. *P001_interview01_EN_transcript.pdf*

# Introduction to Interview – Not recorded

## Background

**Review with participant prior to recording – summarize, don’t read. This should be a casual conversation.**

Thank you for taking the time to speak with me today. Before we can start with the interview let’s go over the information for the study.

We are talking to people such as yourself with Rheumatoid Arthritis (RA). The purpose of our conversation today is to better understand the experience of RA, how it affects you, and whether the new digital outcome measure adequately reflects your personal experiences.

It is expected that approximately 40 participants with RA will be enrolled in this interview portion of the study. There are no treatments being tested in this study.

You are being asked to take part in one interview, which will be audio and screen recorded to ensure we capture everything you say accurately. The interview is expected to take about 2 hours to complete. The recording of the interview will be transcribed but no names will appear in the written transcript. All your responses will be kept confidential and deidentified; your name will not be linked with any of your responses for data analysis. Recording the interview is a required part of the study. If you do not want to be recorded, you may not take part in the study. If you wish, you may choose to allow your video recording to be shared in the future with other researchers for training or research purposes. In this case, a recording of your voice and the mapping process on the screen might be shared however, your other personal identifying information will NOT be shared.

***Sharing your video is completely optional and you are not required to share your video to be part of the study.***

Your participation is voluntary, which means that you do not have to take part in the interview. You can skip any question you do not want to answer, and you can choose to stop the interview at any time.

**Before we proceed, do you have any questions?** (Address questions before proceeding.)

**Is it okay for me to record the conversation today?** (this will be restated for recording)

***If yes, continue to “Background for All Interviews.”***

***If no:*** Unfortunately, since you do not agree to the recording of this session, you won’t be able to participate in this study. Thank you for your willingness to consider participation in this study.

## Interviewer role

My role here is to ask questions and to listen. I will ask questions about your experience and I will move the discussion from one question to the next to try to keep us on track so that we can finish on-time.

I am not your medical doctor, so I am not able to give medical advice. I encourage you to follow-up with your regular doctor if you have any questions about your condition after this interview.

Please feel free to let me know if you need a break. You can ask me questions at any time.

## Recording begins

| **Begin recorder here:**  Start Zoom recorder, start 2 digital voice recorders as back up if Zoom fails.  This is participant ID *[****insert ID number here****]* for the RA Study on *[****Date, time****].*  State interviewer initials.  Do I have your permission to record this interview? ***Verbal response required.***  Can you confirm you read and signed the Informed Consent Form for the study? ***Verbal response required.***  Do you wish to have your audio/video recording shared with others for future training or research purposes? |
| --- |

# Section 1. Personally Important Symptoms


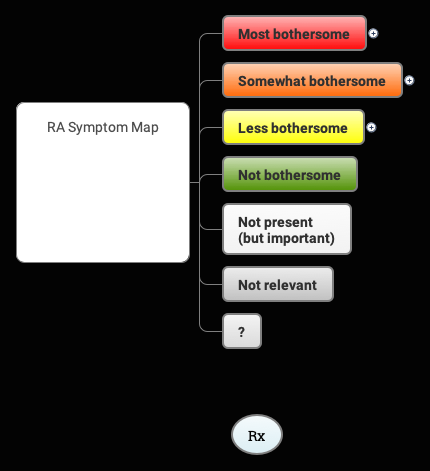


I'm going to ask you to describe your symptoms and how they affect you. We will use this information to create a map or a "picture" of symptoms that are important to you, based upon what you tell me. (Map will be pre-populated with symptoms from the symptom survey)

*[The interviewer will demonstrate technique and check:*

*Screen is at a comfortable reading size*

## In-depth review of RA symptoms

**1. Let’s talk about your symptoms of RA and how these affect you (physically or psychologically).**

[**Step 1 of the symptom mapping activity begins here.** As the participant directs, the interviewer will map the participants symptom experiences. The map will have some symptoms prepopulated from the symptom survey, but new symptoms can be added as they arise during discussion. Most participants will start with what bothers them most or from top down in the list, but any order is permissible. The interviewer should start open – allowing the participant to lead with what is natural to them, but should be sure to systematically evaluated for all a priori symptoms from the symptom check list.]

Possible probes:

- - - - What do you experience? (explore characteristics of symptom)
      - How does it affect you? / How does it make you feel?
      - What do you do when it happens? Do you do anything differently as a result?
      - Does it affect your ability to do things? (limitations or compensating behaviors)
      - What makes it bothersome to you (or not bothersome)?
      - Do you want to add anything else to this before we move on?

**[close symptom and move to next]**

*Symptoms may be reorganized and grouped based on the participants perspective.

## Ranking of symptoms by bothersomeness

**2. Now, let's reorder your symptoms based on how important they are to you. [symptoms collapsed]**

*[The interviewer will rank the symptoms by bothersomeness, hierarchically, calling out symptoms that are particularly important to the individual with boundaries or summary brackets. Symptoms should be in fully collapsed state for this.]*

# Section 2. Patient Perspectives on DHT – Debriefing on sensors

In this study, you wore passive monitoring sensors to measure your RA symptoms. The goal of the following questions is to determine if these sensors capture what matters to you about RA. We will ask you the same questions about each of the main items the sensors are designed to measure.

*Start with the DHT tab. Attach concise answers to the DHT concepts and hide when done (collapse).* ***TIPS****: drill down to manage screen space – make sure the DHT is in logic chart right format. Call out relevant items with a boundary.*

##
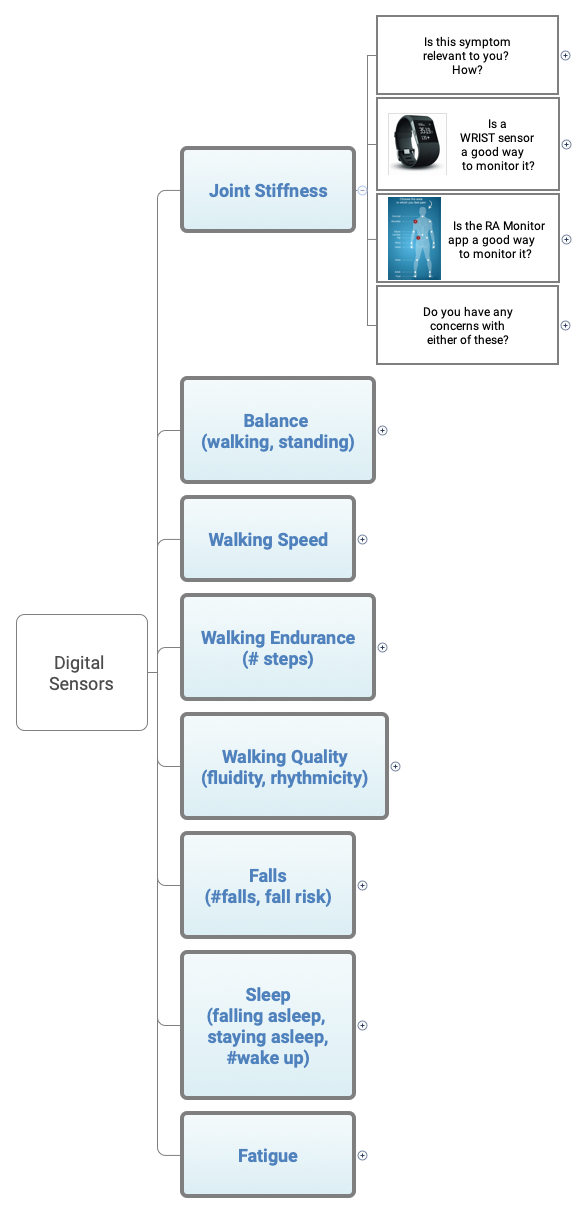
Standardized questions for digital measures.

**3. You wore a [sensor on your wrist], which looked like this (show image of sensor). The [sensor] measured [describe symptom measured and how it is captured].**

**3A.** Is the symptom measured by the sensor relevant to you personally? (Yes/No/Not sure, Plus: Explore how it is or is not relevant) *Highlight relevant items with boundary to call out.*

- - - - Does this symptom relate to something that you personally experience with RA **or**
      - Does it relate to a symptom of RA that you feel is important to monitor?

Sensor concepts for debrief:

- Joint Stiffness
- Balance (walking, standing)
- Walking speed
- Walking endurance (# steps)
- Walking quality (fluidity, rhythmicity)
- Falls (#falls, fall risk)
- Sleep (falling asleep, staying asleep, #wake up)
- Fatigue
  - - - (Identify which personally important symptom the measure relates to if unclear from discussion.)

**3B.** Does wearing a sensor on the [**wrist**] seem like a good way to monitor this symptom in RA?? (Why/why not?)

**3C.** Does wearing a sensor on the [**back**] seem like a good way to monitor this symptom in RA?? (Why/why not?)

**3D.** Did you have any other concerns about either of these measures? (safety, tech, other problems)

**Repeat this process for each symptom concept that the sensor is designed to monitor.**

1. **Are there important symptoms of RA that these symptoms don’t address?**
   - What would you want to see included in future assessments?

# Section 3: Mapping DHT to Personal RA Symptoms

Now let's incorporate each of the items we just talked about into your personal symptom map *. All items can be moved at the end of the debriefing section. Digital sensors should be in collapsed state so that only the item shows, not the details. This will help with managing the amount of information on the screen. Symptom map should also be in collapsed state. Relevant items can be selected all at once using the “multi select” function, and same approach for the not relevant items, to minimize jumping around.*

## Integrate PRO Pictographs into the symptom map

**5. Confirm placement of the digital sensors in the map relative to personal symptoms.**

- - - 1. Items that related to personal experiences should be attached to those experiences in the map.
      2. Items that were deemed relevant but not related to any personal experiences are "no current issues but important to monitor for"
      3. Items of questionable relevance are placed in the "not important or not relevant" category and additional details may be added to explain lack of relevance.

# Closing questions

**6. How did you feel about the symptom mapping we did to today to describe your symptoms?**

- 1. What did you like or dislike about it?
  2. Are there any changes you think we should make to this technique?

**7. Do you have any other thoughts or comments?**

**8. Do you want a copy of your map emailed to you?**

Thank you for your time and for all the insightful information and experiences you have shared with me today. Now, let’s discuss the next steps before we end the interview*.*

**STATE FOR RECORDING: This concludes the interview and the recording.**

**[Stop recording and go through any closing logistical items with the participant.]**


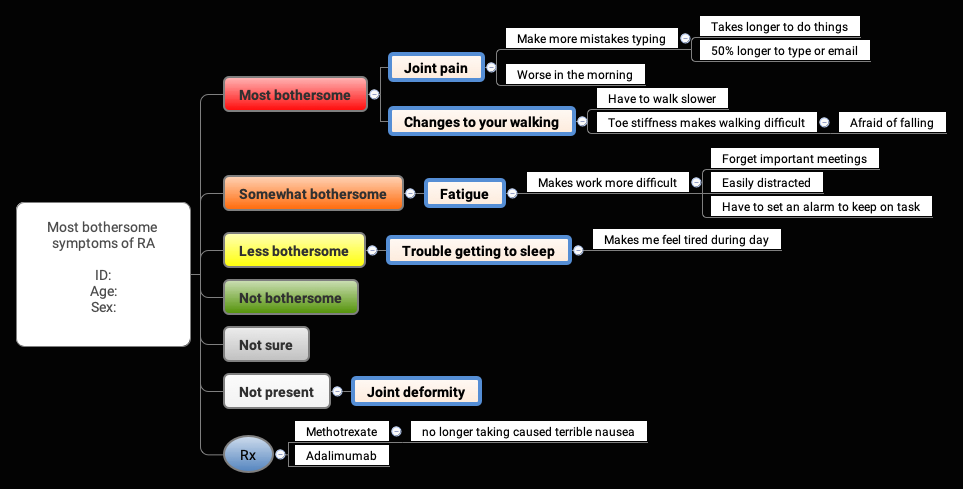
Example of patient map:

Rheumatoid Arthritis Symptoms for Pre-interview Survey

Please see pre-interview survey for symptoms will be assessed *a priori* as starting point for map. This will be done using a checklist (present not present) with open response items to describe what symptoms are most bothersome. This is then used to start a baseline map and ensure systematic exploration of symptoms during the interview.

**References:**

Deane, Kevin D., and V. Michael Holers. “Rheumatoid Arthritis Pathogenesis, Prediction, and Prevention: An Emerging Paradigm Shift.” *Arthritis & Rheumatology (Hoboken, N.J.)*, vol. 73, no. 2, 2021, pp. 181–93, https://doi.org/10.1002/art.41417.

Li, Chun, et al. "Management of Rheumatoid Arthritis with a Digital Health Application: A Multicenter, Pragmatic Randomized Clinical Trial." *JAMA Network Open*, vol. 6, no. 4, 2023, p. e238343, [https://doi.org/10.1001/jamanetworkopen.2023.8343. Accessed 9 Jan. 2025](https://doi.org/10.1001/jamanetworkopen.2023.8343.%20Accessed%209%20Jan.%202025).

Misra, Durga Prasanna. “Clinical Manifestations of Rheumatoid Arthritis, Including Comorbidities, Complications, and Long-Term Follow-Up.” *Best Practice & Research. Clinical Rheumatology*, 2024, pp. 102020-, https://doi.org/10.1016/j.berh.2024.102020.

Section B. Perspective Mapping Implementation Checklist

| ✔ | Planning and Preparation |
| --- | --- |
|  | Develop the approach, including interview guide, map and any other materials. |
|  | Get participant feedback on approach and materials – consider an advisory board. |
|  | Pre-test the interview process multiple times. |
|  | Decide whether to use one or two interviewers. |
|  | Establish pre- and post-interview procedures including semi-structured interview guide. |
|  | Develop pre- and post- interview email scripts to participants (invitations and thank you). |
|  | Have a systematic training plan – be fluent before going live. |
|  | PRACTICE – PRACTICE – PRACTICE. |
|  | Record and debrief on practice sessions. Review practice videos to improve technique. |
|  | Assess the visual impact of activity on screen from the participants perspective. |
|  |  |
| ✔ | prior to the interview |
|  | Send the participant a reminder. |
|  | Provide alternate contact information to participants for technical issues. |
|  | Check batteries on backup recorders. |
|  | Turn on Xmind “autosave” to prevent data loss if software crashes. |
|  | Edit system settings in Zoom to set preferences for video recording during screen share. |
|  | Set up the baseline map if using an *a priori* approach. |
|  | Double check all equipment and procedures prior to each interview. |
|  | Arrange interview guides or other study materials on screen. |
|  | Pay attention to ergonomics. |
|  |  |
| ✔ | During the Interview |
|  | A/V Record the entire interview with screen sharing. |
|  | Save A/V recording for validation and coding. |
|  | Run two back up audio recorders. |
|  | Monitor the visual experience of mapping for the participant. |
|  | Make sure on-screen activity is not distracting or overwhelming. |
|  | Offer breaks frequently, especially if signs of participant fatigue are evident. |
|  | Listen, clarify, distill, transcribe, organize data in the map. |
|  | Stay true to participant voice. Paint a picture of the experience using their words. |
|  | Sort and categorize concepts into the QT framework. |
|  |  |
| ✔ | Immediately After the interview |
|  | Label and store all files using the designated naming convention. |
|  | Make PDF copies of all outputs immediately following the interview to prevent data loss. |
|  | Assess all files for integrity and readability prior to storage. |
|  | Keep backup copies. |
|  | Email copies of maps to participant for reciprocity. |
|  | Review the interview videos to promote continuous quality improvement. |
|  | Start data analysis. |
